# Gruel Creep Feeding Accelerates Growth and Alters Intestinal Health of Young Pigs

**DOI:** 10.3390/ani12182408

**Published:** 2022-09-14

**Authors:** Timothy E. Boston, Feng Wang, Xi Lin, Suzanne Leonard, Sung Woo Kim, Denny McKilligan, Vivek Fellner, Jack Odle

**Affiliations:** 1 Department of Animal Science, North Carolina State University, Raleigh, NC 27695, USA; 2 TechMix Global, Stewart, MN 55385, USA

**Keywords:** creep feeding, intestinal health, piglet growth, weaning

## Abstract

**Simple Summary:**

The weaning period is one of the most stressful periods in a piglet’s life due to abrupt changes in diet and environment. To mitigate production loss, creep feed is given to piglets to supplement sow’s milk, but the intake of typical dry creep feed is low. Alternatively, liquid diets presented as a gruel may have increased positive effects post-weaning. The objective of this study was to discern whether a gruel pre-weaning supplementation could better prepare piglets to handle increased stress encountered after weaning. We conclude that piglets fed gruel creep feed had greater feed intake and body weight than pigs that had no supplementation, and the effects were sustained through the first week of weaning.

**Abstract:**

To combat the stress of weaning, we utilized novel gruel creep feeders to supplement suckling pigs with divergent soluble (*n* = 6 litters) versus insoluble (*n* = 6) diets compared with un-supplemented controls (*n* = 6). Post-weaning, pigs were fed a common phase 1 diet. Average daily weight gains of pigs fed soluble and insoluble creep diets were 53% and 17% greater than control pigs, respectively (*p* < 0.01). Creep intake was higher (82%) for pigs fed the soluble diet, and the accompanying weight increase was sustained post-weaning (*p* < 0.02). Villus measures were prematurely altered in soluble-creep-fed pigs (*p* < 0.01), with decreases in villi length, crypt depth, and villus area pre-weaning. No effects of treatment were detected for VFA concentrations and pH in the cecum. There was an interaction between treatment and age for several pro- and anti-inflammatory cytokines (*p* < 0.01), where soluble-creep-fed pigs had increased cytokine levels with age, whereas cytokine levels in the insoluble and control groups decreased over time. We conclude that a soluble creep diet fed in a gruel state during the pre-weaning period has a positive impact on weaning weight that is sustained post-weaning, and is accompanied by alterations in the intestinal health of young pigs.

## 1. Introduction

At weaning, piglets exhibit decreased feed intake, leading to stagnation in body weight gain. The shift from a highly digestible milk diet to a fiber-rich solid feed negatively impacts intestinal health measures, such as shortening of villi and increase in crypt depth [1,2]. Supplementing with a milk replacer can increase pre-weaning weight gain [3,4,5], but does not remedy the post-weaning decline in piglet performance. To help mitigate post-weaning stressors, creep feeding during the lactation period has been a practice utilized by the swine industry to provide nutrients to young pigs and to promote their adaptation to solid feed [6,7,8]. While there is a dearth of evidence to prove that creep feeding definitively increases weaning weight, Byrgesen et al. [9] reported effects related to gruel versus dry creep feeding. Building upon this, and as technology advances, new delivery methods of creep feed are being developed, varying from behavioral stimulation to easily accessible feeder designs [10,11]. Generating a larger percentage of creep feed “eaters” benefits entire litters, easing overall weaning stress. While most studies are either strictly lactation or strictly nursery studies, fewer studies span both periods [12]. Even with some experiments following pigs through long-term creep studies, there are few conclusive agreements on the long-term effects. Nutritional intervention is important for minimizing weaning stress, and the development of new creep feed methods is crucial [13]. Furthermore, creep feeding and how it impacts gut biodiversity, fermentation, and immune response all may influence how weaning impacts GI tract health [14,15,16].

The aim of this study was to determine whether novel gruel creep feeders could adequately deliver two divergent feeds (one highly soluble and one insoluble) to suckling piglets, and whether this would have a positive effect on weight gain during lactation that would carry forward into the nursery post-weaning. Additionally, we wanted to determine whether either creep diet would alter intestinal parameters to better prepare piglets for the nursery and mitigate post-weaning stress.

## 2. Materials and Methods

### 2.1. Animals and Experimental Design

The experiment was conducted at the North Carolina State University Swine Educational Unit during August–September 2021. All animal procedures were approved by the Institutional Animal Care and Use Committee.

A soluble creep diet was formulated using mostly soluble ingredients, while the insoluble diet contained ingredients typical of a phase 1 nursery diet (Table 1). The diets contained no antibiotics or growth promoting minerals, and represented extremes in formulation to investigate a full range of piglet responses and to compare feeder delivery of such divergent diets. The diets were not formulated as complete feeds for suckling pigs, but rather as supplements to sow’s milk. Both introduced the pigs to non-milk ingredients in advance of weaning, with the soluble diet containing 20% oatmeal and 10% soy protein isolate. As such, they were designed practically and intentionally to vary not only in solubility but also in nutrient composition and digestibility. Gruel creep feeders (Supplementary Figure S1. Mini transition feeders GE Baker Company, Bury Saint Edmunds UK) were utilized, and they employed an auger that metered the dry diets down into a holding pan where it was mixed with water. Roughly 1.2 kg of feed could be held in the feeder hopper at once, being manually refilled when levels were low. A sensor 80 mm above the plate detected when gruel reached maximum level, upon which it would turn the flow of gruel off. Two dials controlled the water output and time lapse between feeding cycles. The former was set to the highest output, where water was mixed with each creep diet so that there were 18% solids delivered. The latter dial was set for gruel to refill immediately if the sensor detected low levels (ad libitum). Feeders were cleaned and serviced twice a day. Each diet was fed to six litters, and an additional six control litters did not receive any creep feed (*n* = 18 litters total). Multiparous sows (≥parity 2; Smithfield Premium Genetics; Yorkshire × Landrace × Duroc) were randomly assigned to treatment as they were placed into farrowing crates on D109 of gestation. Farrowing was induced with PGF2α injected on gestation D112. Cross-fostering of piglets was minimized but was used to ensure at least 10 pigs/litter by day three of age. On D7, a total of 196 pigs (average of 10.8/litter) began the study with an average weight of 2.48 ± 0.39 kg, receiving one of the three treatments. Treatments continued until pigs were weaned at 23 days of age. At weaning, littermates remained intact, with each occupying one nursery pen (1.8 × 1.6 m). All pigs received a common pelleted phase 1 nursery diet of identical composition to the insoluble creep diet (Table 1), and their post-weaning growth was recorded until D31.

Twelve video cameras (four GoPro HERO 8, eight AKASO Brave 7) were mounted above each feeder, capturing timelapse video at one frame per second at a resolution of 1920 × 1080 pixels. On the first day of treatment, selected pigs were marked with livestock paint colors and patterns to allow for individual pig identification in the videos. Amount of time at feeders from 09:00 to 14:00 on the observation days was measured in seconds. A piglet was labeled as feeding in a video frame when their nose or head was observed to be partially or completely in, above, or touching the creep feeder. Piglets that met these criteria but were clearly lying down were not counted as feeding. Feeders and creep feed were visible in videos, therefore, observers were not blinded to treatment, although they were blinded to pen number. At four days prior to weaning, chromic oxide also was added to the creep diets (3 g/kg) to serve as a qualitative fecal marker of creep intake along with camera footage [17].

At one day prior to weaning (D22 of age) and one week post-weaning (D31 of age), one pig per litter (*n* = 36 total) was euthanized by AVMA-approved electrocution for measurement of intestinal parameters. Pigs of median weight were selected, and pre-weaning pigs displaying green fecal swabs and/or video “high time at feeder” were chosen. Immediately prior to euthanasia, blood was collected via jugular venipuncture (K2-EDTA Vacutainers) and centrifuged, and plasma was frozen for subsequent analysis.

Following exsanguination, a midline incision was made to remove the intestinal tract. Weights of small (duodenum–ileum) and large bowels (cecum–anus) were measured and expressed as a relative percentages of body weight. Additional sampling included ileal contents, ileal mucosa, ileal subsection fixed in 10% formalin for histology, fresh cecal pH, and colon digesta. All samples except for formalin-fixed subsections were frozen in liquid nitrogen and stored at −80 °C.

### 2.2. Cytokine Analysis

Plasma was analyzed on a Porcine Cytokine/Chemokine 13-Plex Discovery Assay^®^ Array (PD13, Eve Technologies, Calgary, CA, USA). Specific cytokines measured (pg/mL) included GM-CSF, IFNγ, IL-1α, IL-1RA, IL-1β, IL-2, IL-4, IL-6, IL-8, IL-10, IL-12, IL-18, and TNFα.

### 2.3. Histology

Ileal subsections were fixed in 10% formalin for 24 h, before a 70% ethanol wash, and then stored in 70% ethanol. Samples were then dehydrated, embedded in paraffin, cut into 5 μm-thick cross-sections, and mounted on polylysine-coated slides. Slides were stained with hematoxylin and eosin and sealed. Ten images per slide were captured at both 4x and 10x magnification on an Olympus CX31 Light Microscope (Lumenera Corporation, Ottawa, Canada) with an Infinity 2-2 digital CCD camera to measure villi length, crypt depth, and villus area. Measurements of villi and crypt depth were calculated as described by Moita et al. [18], while villus surface area was calculated as described by Hess et al. [19].

### 2.4. Gas Chromatography

Frozen cecal contents were thawed on ice, and 1 mL of 0.5 N HCL was added to the tube. Samples were vortexed and incubated at room temperature for 24 h. Samples were then centrifuged at 900× *g* for 10 min to separate the supernatant. One mL of the supernatant and 200 μL of metaphosphoric acid (MIS) were transferred into an Eppendorf tube and centrifuged at 32,000× *g* for 10 min. As an internal standard, MIS was prepared by weighing 25 g of metaphosphoric acid into a beaker and dissolving in 50 mL of deionized water. Subsequently, 0.2 g of 2-ethylbutyric acid and another 25 mL of water was mixed in the beaker, bringing the final solution to 100 mL. This “cleaned” supernatant was pipetted into a GC vial for analysis on a Varian gas–liquid chromatograph (model CP 3380/3800). The column (NUKOL Fuser Silica Capillary Column) measured 30 m in length × 0.25 mm i.d., and 0.25 μm film thickness, reaching a maximum temperature of 300 °C [20].

### 2.5. Statistics

Creep treatment effects on growth performance data were analyzed using the general linear model procedure of SAS (Cary, NC, USA) appropriate for a completely randomized design, with litter as the experimental unit. Intestinal data were analyzed according to a 2 × 2 factorial design with creep treatment and piglet age (d22 versus d31) as main effects. Least square means were separated using a protected least significant difference test. Differences were noted when *p* < 0.05 and trends identified when 0.05 < *p* ≤ 0.1.

## 3. Results

### 3.1. ADG and Feed Intake

During the first week of creep feeding (D7–14), pigs receiving the soluble creep diet grew 44% faster (*p* < 0.01) than controls and pigs fed the insoluble creep diet (Table 2). During the second week (D14–23), both insoluble- and soluble-creep-fed groups grew faster than control litters (*p* < 0.01) by 32 and 62%, respectively, with performance of soluble-creep-fed pigs remaining greatest. The accelerated growth of litters fed soluble versus insoluble creep feed were associated with 200% (D7–14) and 175% (D14–23) increases in creep feed consumption (*p* < 0.01) on a per litter basis. Similar differences were observed when expressed on a per pig basis. Soluble-creep-fed pigs were observed at the feeders an average of eight times higher than insoluble-creep-fed pigs at D14 (*p* < 0.033; Supplemental Figure S2), but no difference was detected between groups at D23. The accumulative effects by the end of lactation (D23) were that average piglet body weights increased progressively (*p* < 0.003) from control (5.51 kg/pig) to insoluble-creep-fed (6.33) to soluble-creep-fed pigs (7.65). During the first week post-weaning (D23–31), no differences were detected (*p* > 0.1) in growth or feed intake on a per litter basis, with noted larger variation (Table 2). The gain:feed ratio was only calculated during the first week of nursery because piglet milk consumption was not measured during lactation. Post-weaning, control pigs had higher efficiency (*p* < 0.045) than pigs fed the soluble creep diet. Despite this, bodyweights of pigs fed soluble creep feed during lactation remained 21% greater (*p* < 0.017) than control and insoluble-creep-fed groups at one week post-weaning (D31).

### 3.2. Intestinal Measures

Cecal pH was on average 0.5 units lower (*p* < 0.01) in pigs after weaning (5.69) than before weaning (6.20), and tended (*p* = 0.055, treatment main effect) to be lower in pigs fed the insoluble creep diet (Table 3). The relative weight of the small intestine expressed as a percentage of body weight increased with age (*p* < 0.01), and the increase was greatest for pigs fed the insoluble creep diet (Treatment x Age interaction, *p* < 0.01). The relative weight of the large intestine also increased with age (*p* < 0.01), but no differences among treatments were detected (*p* > 0.1).

Villi length was 38% (*p* < 0.01) and 23% (*p* < 0.01) longer on D22 for control and insoluble creep diet pigs (respectively) than in pigs fed the soluble diet (Table 4). At D31, villi in the pigs fed the insoluble creep remained 27% (*p* < 0.01) longer than in pigs fed the soluble creep diet. There was an effect of age (*p* < 0.03) on the length of villi, increasing by 12% from D22 to D31. At D22, there were no effects detected on crypt depth, but at D31, crypt depth increased in pigs fed the control and insoluble diets by 26% and 38%, respectively (*p* < 0.001), compared to pigs fed the soluble diet. Crypt depth also increased by 33% from D22 to D31 (*p* < 0.01). The villi:crypt ratio on D22 was different (*p* < 0.01) between the pigs fed the control versus the soluble creep diets (3.01 and 2.34), but no difference was detected in ratio in pigs fed the insoluble diet compared with those fed the other diets. At D31, there were no effects detected in terms of treatment on villi:crypt ratio, but there was an overall effect of age (*p* < 0.02) and an interaction trend between treatment and age (*p* < 0.08). The villus area on D22 was significantly different (*p* < 0.001) in pigs fed the control and soluble creep diets, but pigs fed the insoluble diet were again not found to be different than the other treatment groups. At D31 the villus area in pigs fed both the control and insoluble diets were 15% and 14% greater than in pigs fed the soluble diet (*p* < 0.03). There was a positive trend with the effect of age, with villus area increasing by 25% and 35% for both insoluble and soluble diets, respectively (*p* < 0.09).

### 3.3. VFA

Volatile fatty acid (VFA) analysis (Table 5) revealed no detectable interactions between treatment and age (*p* > 0.1). However, the molar proportion of acetate increased with age (*p* < 0.01), with the biggest increase (27%) observed in pigs fed the soluble diet (*p* < 0.01). Branched-chain acids decreased with age (*p* < 0.01) and differed among treatments (*p* < 0.03). There were no effects detected among propionate, butyrate, and valerate (*p* > 0.1) for either treatment or age. For total VFA concentration (mM), there was a trend (*p* < 0.09) in terms of increasing amounts of VFAs in the cecum with age, but no effects of treatment were detected.

### 3.4. Cytokines

**Figure 1:**
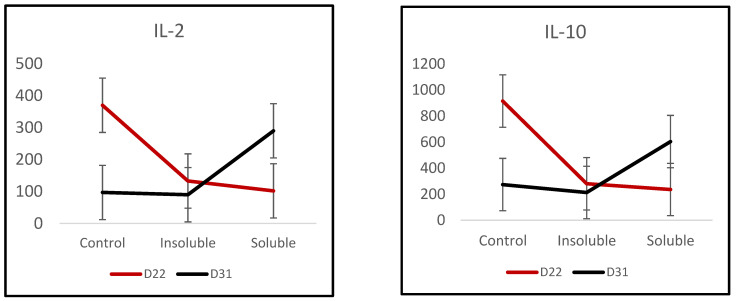
Plasma IL- 2 and IL-10 concentrations (pg/mL) in pigs fed insoluble or soluble gruel creep diets, measured before (D22) or after (D31) weaning, compared with non-creep-fed controls. Significant effects of diet and age were not detected (*p* > 0.1). Treatment by age interaction (*p* < 0.05). Similar interactions were observed for IL-1α, IL-1β, IL-4, IL-6, and IL-18 (see Supplemental Table S1).

There were no main effects of treatment or age detected on plasma cytokine concentrations (Supplemental Table S1), but several cytokines displayed interactions with treatment and age (Figure 1). Both the insoluble and soluble diets had significantly lower levels of IL-1α, IL-1β, IL-2, IL-4, IL-6, IL-10, and IL-18 compared to the control pigs pre-weaning. However, post-weaning, only pigs fed the soluble diet had increased cytokine levels compared to pigs fed the insoluble and control diets, with interaction *p*-values of 0.015, 0.002, 0.038, 0.026, 0.049, 0.056, and 0.02 for each cytokine, respectively.

## 4. Discussion

Litters were kept intact after weaning, contrary to common commercial practices, to minimize social stress caused by fighting among pigs when litters are mixed. This allowed us to better discern whether creep feeding could better prepare the pigs to adapt to the dietary stressors faced during the first week of weaning.

When employed, intake of typical creep feed is generally low, leading to minimal improvements in weaning weights. Fraser et al. [21] tested low-complexity versus high-complexity creep diets and reported pre-weaning ADGs of 263 g/d and 232 g/d. By comparison, pigs fed our insoluble and soluble gruel creep diets gained 275 and 337 g/d, respectively, inferring that addition of water to the gruel could entice greater consumption and growth. Another case of low creep intake was reported by Cabrera et al. [22], where their creep diets with differing levels of glutamine had intake levels ranging from 45–50 g/pig over the last week of lactation. Our findings show markedly higher creep intake in soluble-creep-fed pigs (106 g/pig) and slightly higher levels in insoluble creep pigs (52 g/pig). Toplis et al. [23] reported weaning weights where gruel-supplemented pigs weighed 6.7 kg compared to our soluble-diet-fed piglets, weighing 7.7 kg. Compared to these previous studies, we observed markedly accelerated intake and growth in pigs supplemented with the soluble creep diet. This response likely stems from greater creep intakes associated with gruel delivery from the novel creep feeders. In both Middelkoop et al. [10] and Sulabo et al. [11] studies, the design of the feeders had measurable effects on post-weaning feed consumption and proportion of eaters, respectively. Middelkoop used “play feeders”, where piglets ate from a foraging-stimulating feeder in comparison to a conventional round plate. Using piglets’ natural curiosity and foraging instincts is a creative way to stimulate intake of non-milk feed at an early age. While there were no effects detected of feed intake in lactation, there was increased post-weaning feed intake and reduced diarrhea. In the Sulabo et al. study, a creep feeder was used in which a rotary hopper dispensed feed ad libitum and minimized waste. They found that the rotary feeder with the hopper had significantly less feed disappearance relative to a rotary feeder without a hopper and a pan feeder. On the other hand, the proportion of eaters was higher for the rotary feeder with the hopper. While the previous studies involved dry creep feed, our study tested gruel feed, providing superior feed physical state (gruel) paired with an innovative feeder design.

Litters supplemented with soluble creep feed during the second and third weeks of lactation displayed substantially accelerated growth over non-creep-fed control litters during both weeks of supplementation. In contrast, litters given insoluble creep feed only showed improved performance during the third week of lactation. The difference between the diets was driven by greater intake of the soluble creep diet. This could potentially be attributed to the addition of sweeteners to the soluble diet compared to the insoluble diet, although Figueroa et al. [24] found that there were no differences in feed intake detected based on flavor preferences. Another reason for high creep intake might also be season dependence. Azain et al. [25] described a strong seasonal impact on supplemental milk replacer intake based on winter versus summer lactation periods. The increase in piglet intake of supplemental milk was driven by heat stress, with sows eating less and producing less milk during the summer compared to winter. Because our study took place during late August–September, this would likewise result in high supplemental creep intake, in agreement with Azain et al. It is important to note that we would need more data to confirm this seasonal effect, as well as to confirm whether the seasonal effect would be the same for gruel creep feed. We can thus infer that it is composition of the creep diet that drove the differences in feed intake.

The insoluble diet had less of an impact on body weight and weight gain potentially due to an inability to mix uniformly with water. We observed that the soluble diet mixed better with the water compared to the insoluble diet, leading to the increase in feed intake described above. Studies by Kim et al. [26] and Price et al. [27] reported an increase in the consumption of liquid diets compared to a post-weaning dry nursery diet. Price et al. found that pigs exhibited higher consumption of liquid diets through the first two weeks of weaning compared to dry-diet-fed pigs. Kim et al. studied piglets from 14 days of age to market weight, and found that pigs fed liquid feed in early life reached market weight four days sooner. Both studies support liquid feeding in comparison to dry feeds, congruent with the idea that young pigs are behaviorally inclined to consume liquid feed. Consistent with previous studies, we also detected a sustained nursery feed intake and increase in weaning weight due to gruel creep feeding. The physical form of diets altering morphological traits was also observed by Cappai et al. [28,29]. Mandibular glands and mucosal thickness of the terminal ileum in young pigs were both effected by coarseness of feed, both increasing in size and thickness with increased particle size. While solubility of diets contributed, in part, to higher feed intake and increased weight gain, the stark difference in composition likely contributed as well. The divergent diets were purposefully designed to represent extremes in formulation that could be utilized with the gruel feeders. The soluble diet contained highly digestible ingredients, for example, being higher in whey permeate, lactose, and SID lysine; whereas the insoluble diet contained less-digestible ingredients but represented the precise phase 1 diet the pigs were fed post-weaning.

Even though there was a decrease in post-weaning performance exhibited by the soluble and insoluble groups, the impacts were blurred by the high degree of variability. We noted high variation in feed intake, especially in pigs that had been fed the soluble creep diet during lactation. Though not statistically significant, growth was numerically lowest (79 g/d) for pigs fed the soluble diet during lactation compared to control (153 g/d) or insoluble (100 g/d) groups. Furthermore, the improved gain:feed ratio of control pigs over pigs fed soluble creep illustrated compensatory performance. Despite this lower growth rate, pigs fed the soluble creep retained the greatest body mass at the end of the growth trial. Results from other studies vary as to whether pre-weaning body weight differences are sustained over time. Christensen et al. [30] concluded that providing supplemental nutrition such as creep feed does positively affect weaning weights, but this gain was lost over time in the nursery. On the other hand, Byrgensen et al. [9] reported a tendency for dry-creep-fed piglets during lactation to have improved weight gain in the nursery. Further studies using improved creep feeder designs need to be conducted to determine whether the weight gain advantage during lactation is sustained throughout the nursery phase.

Our collective observations regarding the insoluble diet are that pigs consumed more during the third week of lactation than during the second week, leading to improved weight gain over control litters. While this growth advantage was no longer detected in the nursery, pigs fed the insoluble creep diet showed slightly lower cecal pH, suggesting increased fermentation. A greater increase in the relative weight of the small intestine may also suggest that insoluble diet pigs were responding to diet composition. The increase in small intestine weight may be an indication of higher feed retention in the digestive tract. In comparison, the soluble-creep-fed pigs had the smallest intestinal weights.

Villi height and crypt depth of the control pigs were hypothesized to be the highest, since they only had highly soluble sow’s milk to digest. Piglets have the longest villi at pre-weaning compared to the post-weaning phase due to multiple stressors encountered including the abrupt diet change [31,32,33]. Increased crypt depth is known to be indicative of increased cell proliferation. This is generally higher when there is more cell regeneration and maturation of the intestinal lining, which occurs when eating a more complex diet. It is not surprising, then, that all diets had increased crypt depth one week post-weaning due to stressors of the change from lactation to the nursery. The soluble diet displaying the shortest villi length of any treatment post-weaning was surprising, but this may be explained by the significantly increased creep feed consumption. Compared to the sow’s milk control treatment or the low-intake insoluble diet, the soluble diet was consumed readily, and the non-milk ingredients may have challenged villi morphology at an earlier stage prior to weaning. Van Beers-Schreurs et al. [1] found that villus atrophy was more due to increased levels of feed intake than to the composition of the diet, which supports our findings. Their findings also supported the increased villi height of a milk-predominant diet, hence the longest villi being measured in the control piglets. Both villi:crypt ratios and villus area are consistent with the literature [2]. A 3:1 ratio of villi to crypt depth is common, but the decreased ratio is due to post-weaning decreases in villus length and increase in crypt depth.

Concentrations of VFAs reflect microbial fermentation, and both our insoluble and soluble creep diets provided fermentable ingredients. Early studies, such as that by Holtug et al. [34], examined VFA concentrations in different regions of the digestive tract. They found that VFA concentrations tended to be higher in the large intestine; thus, we obtained digesta samples from the cecum for analysis. We observed that the majority of VFAs were not affected by treatment or age (*p* > 0.1), but acetate and BCAs tended to increase and decrease in percentage, respectively. The decrease in pH in the cecum across all treatments over time was consistent with the increase in VFA production. The review by den Besten et al. [35] proposed that an increase in fiber in pig diets may increase VFA concentrations in the cecum. The increase in fiber reported in our study could be from either corn or oatmeal in the insoluble and soluble diet, respectively. Alternatively, stressors of weaning along with the change in diet possibly altered the microbial environment, significantly changing the percentage of VFA concentrations. Wang et al. [36] found that probiotics altered concentrations of VFAs in the colon, in turn, effecting species richness of microbiota alpha diversity. While there were no specific bioactive ingredients in our diets, changes in diet composition may have altered microbiota concentrations. Further microbiota analyses need to be conducted to verify these results.

Immune responses may be closely tied to microbial communities within the intestine. Duarte et al. [37] reported that microbiota–host interactions modulated the immune system, while the immune system also helped modulate microbiota. Both are closely tied to the other, with changes in either having a downstream effect. Zhang et al. [16] studied immune parameters in the small intestine and how they reacted to differing levels of protein. They found that lower crude protein (CP) levels changed specific bacterial communities and modified mucosal immune parameters. In addition to CP, differing levels of whey permeate for insoluble and soluble diets may have impacted the bacterial communities. This may explain the interaction of treatment and age that we found in soluble-creep-fed pigs and controls. Whether an increased or decreased immune response post-weaning is detrimental to piglets is controversial. More analyses of immune pathways and how they affect weight gain or feed intake of weaned pigs are needed.

## 5. Conclusions

The soluble creep diet formulation provided superior growth performance of suckling pigs, and their heavier weaning weights were maintained through the first week of the subsequent nursery period, despite high variability of intake and growth post-weaning. The insoluble creep diet provided a mild indication of potentially increased fermentation, which could improve weaning transition. High gruel creep feed intake in soluble-diet-fed pigs led to pre-weaning changes in intestinal morphology. Overall effects of diet solubility are tied to composition and digestibility, which also play an important role in the observed increased weight gain and feed intake. For future diet considerations, perhaps slightly more insoluble (fibrous) ingredients could be added to a largely soluble base of ingredients, yielding a formulation intermediate between the soluble and insoluble formulations used in the present study. Alternatively, the inclusion of soluble fiber sources into the soluble formulation may yield desirable results for post-weaning gut health and growth.

## Figures and Tables

**Table 1:** Divergent diets given to suckling pigs using gruel creep feeders.

Ingredient (%)	Insoluble ^1^	Soluble ^2^
Whey Permeate	24.00	38.82
Corn, yellow dent	29.34	−
Soybean Meal	12.00	−
Cookie Meal	10.00	−
Poultry Meal	10.00	−
Fish Meal	4.00	−
Blood Plasma ^3^	4.00	−
Soy protein concentrate ^4^	4.00	−
Poultry Fat	1.00	−
Soy protein isolate	−	10.00
Nonfat Dried Milk	−	26.34
Feeding Oatmeal	−	20.00
Dextrose	−	2.23
L-Lys ^5^	0.52	0.57
DL-Met ^6^	0.25	0.41
L-Thr ^5^	0.17	0.30
L-Trp ^5^	0.02	0.12
Limestone	0.30	0.40
Salt	0.22	1.0
Mineral Premix ^7^	0.15	−
Vitamin Premix ^8^	0.03	−
Other ^9^	−	0.80
Calculated composition		
DM, %	91.4	95.1
ME, kcal/kg	3465	3132
CP, %	25.17	23.13
Lactose, %	19.2	46.5
SID Lys, %	1.51	1.70
Ca, %	0.85	0.67
*p*, %	0.73	0.68

^1^ The insoluble creep diet was also pelleted and used as the phase 1 nursery diet. ^2^ The soluble diet was formulated and manufactured by TechMix Global (Steward, MN). ^3^ American Protein Corporation (Ankeny, IA). ^4^ CJ Selecta (Araguari, Brazil). ^5^ CJ Bio (Fort Dodge, IA) provided supplemental amino acids. ^6^ Evonik (Essen, Germany). ^7^ Mineral premix included per kg of diet: 33 mg Mn as manganous oxide, 100 mg Fe as ferrous sulfate, 110 mg Zn as zinc sulfate, 16.5 mg Cu as copper sulfate, 0.30 mg of I as ethylenediamine dihydroiodide, and 0.30 mg of Se as sodium selenite. ^8^ Vitamin premix included per kg of diet: 6614 IU of vitamin A as vitamin A acetate, 992 IU vitamin D3, 19.8 IU vitamin E, 2.64 mg vitamin K as menadione sodium bisulfate, 0.03 mg vitamin B12, 4.63 mg riboflavin, 18.52 mg D-pantothenic acid as calcium pantothenate, 24.96 mg niacin, and 0.07 mg biotin. ^9^ Provided as percentage of complete diet: 0.25, sodium aluminosilicate; 0.20, Flav/Strawberry; 0.10, Sucram C-150 sweetener; 0.06, L-isoleucine; 0.03, vitamin E 50% spray dried, 0.0025, vitamin A 1,000,000 IU/GM; 0.0025, vitamin D 1.25%.

**Table 2:** Growth of litters fed either insoluble or soluble gruel creep diets versus non-creep-fed controls ^1^.

	----------------Treatment------------------				
	Control	Insoluble	Soluble	SEM	*p* > F
Number of litters	6	6	6		
Initial Body Weight (kg/pig)					
Day 7 ^2^	2.27	2.51	2.68	0.153	0.213
Day 14	3.63 ^a^	3.85 ^a^	4.62 ^b^	0.262	0.043
Day 23	5.51 ^a^	6.33 ^a^	7.65 ^b^	0.369	0.003
Day 31	6.58 ^a^	7.03 ^a^	8.21 ^b^	0.362	0.017
Average Daily Gain (g/pig)					
Day 7–14	193 ^a^	192 ^a^	278 ^b^	20	0.010
Day 14–23	208 ^a^	275 ^b^	337 ^c^	18	0.001
Day 23–31	153	100	79	25	0.134
Day 7–31	187 ^a^	197 ^a^	240 ^b^	11	0.006
Average daily feed intake (g/litter)					
Day 7–14	0.00 ^a^	143 ^b^	294 ^c^	27	0.001
Day 14–23	0.00 ^a^	533 ^b^	935 ^c^	81	0.001
Day 23–31	1891	1686	1911	216	0.723
Average daily feed intake (g/pig)					
Day 7–14	0.00 ^a^	13 ^b^	28 ^c^	2.5	0.001
Day 14–23	0.00 ^a^	50 ^b^	91 ^c^	7.6	0.001
Day 23–31	186	185	210	15.6	0.460
Gain:Feed					
Day 23–31	0.82 ^a^	0.57 ^a,b^	0.35 ^b^	0.12	0.045

^1^ Litters were fed diets from D7 to D23 of age and compared to control litters receiving no creep feed. After weaning (D23), pigs were fed a common phase 1 nursery diet (Table 1) for one week (until D31 of age). ^2^ Piglet age. ^a,b,c^ Means within a row lacking a common superscript differ (*p* < 0.05).

**Table 3:** Intestinal characteristics in piglets fed insoluble or soluble gruel creep diets, measured before (D22) and after weaning (D31), compared with non-creep-fed controls ^1^.

	------------------------Treatment-----------------------							-----------*p* > F-------------		
Items	Control		Insoluble		Soluble		SEM	Trt ^2^	Age	T × A ^3^
	D22	D31	D22	D31	D22	D31				
Cecal pH	6.33 ^b^	6.01 ^a,b^	6.00 ^a,b^	5.65 ^a^	6.28 ^b^	5.77 ^a^	0.13	0.06	0.01	0.78
Small Intestine (%) ^4^	4.04 ^a^	6.55 ^c^	4.40 ^a^	8.86 ^d^	4.79 ^a,b^	6.06 ^b,c^	0.45	0.01	0.01	0.01
Large Intestine (%)	2.22 ^a,b^	3.75 ^d^	2.38 ^a,b,c^	3.44 ^c,d^	1.85 ^a^	3.57 ^d^	0.61	0.61	0.01	0.50

^1^ One pig per litter was sampled at weaning (D22) or at one week post-weaning (D31). Data are means and SEM, n = 6. ^2^ Treatment. ^3^ Treatment × Age. ^4^ Intestinal weights are expressed as % of body weight. ^a,b,c^ Means within a row lacking a common superscript differ (*p* < 0.05).

**Table 4:** Intestinal morphology in pigs fed insoluble or soluble gruel creep diets, measured before (D22) and after (D31) weaning, compared with non-creep-fed controls ^1^.

	--------------------Treatment--------------------							--------- *p* > F ----------		
Items	Control		Insoluble		Soluble		SEM	Trt ^2^	Age	T × A ^3^
	D22	D31	D22	D31	D22	D31				
Villi (μm)	268 ^a,b^	262 ^a,b^	238 ^a,b^	291 ^a^	193 ^c^	230 ^b^	14.8	0.01	0.03	0.15
Crypt (μm)	90.8 ^b^	123.2 ^a^	90.6 ^b^	134.4 ^a^	84.6 ^b^	97.3 ^b^	6.36	0.01	0.01	0.06
Villi:Crypt ratio	3.0 ^a^	2.16 ^b^	2.65 ^a,b^	2.40 ^b^	2.34 ^b^	2.40 ^b^	0.19	0.54	0.02	0.08
Villus area (μm^2^ × 10^−2^)	776 ^a^	753 ^a^	592 ^b,c^	743 ^a^	482 ^c^	654 ^b^	69.6	0.03	0.09	0.34

^1^ One pig per litter was sampled at weaning (D22) or at one week post-weaning (D31). Data are means and SEM, n = 6. ^2^ Treatment. ^3^ Treatment × Age. ^a,b,c^ Means within a row lacking a common superscript differ (*p* < 0.05).

**Table 5:** Volatile fatty acid (VFA) concentrations (mol%) in the cecum of pigs fed insoluble or soluble gruel creep diets, measured before (D22) or after (D31) weaning, compared with non-creep-fed controls ^1^.

	-----------------------Treatment--------------------							------------*p* > F----------		
Items	Control		Insoluble		Soluble		SEM	Trt ^2^	Age	T × A ^3^
	D22	D31	D22	D31	D22	D31				
Acetate	62.7 ^a,b^	64.8 ^b^	60.4 ^a,b^	68.3 ^b^	52.8 ^a^	67.0 ^b^	3.6	0.41	0.01	0.27
Propionate	15.7	21.4	22.6	24.3	19.8	22.0	2.9	0.26	0.18	0.77
Butyrate	5.4	6.7	5.5	5.7	11.3	5.7	1.8	0.26	0.37	0.16
Valerate	3.0	1.1	1.6	1.1	4.1	2.4	1.1	0.26	0.17	0.80
BCA ^4^	13.2 ^a^	5.9 ^b^	10.0 ^a^	0.55 ^c^	12.1 ^a^	3.0 ^b,c^	1.5	0.03	0.01	0.74
Total ^5^	77.5	117.3	91.3	80.5	81.9	127.0	16.9	0.55	0.09	0.21

^1^ One pig per litter was sampled at weaning (D22) or at one week post-weaning (D31). Data are means and SEM, n = 6. ^2^ Treatment. ^3^ Treatment × Age. ^4^ Branched-chain acids are isobutyrate and isovalerate. ^5^ Totals are concentrations in units of mM. ^a,b,c^ Means within a row lacking a common superscript differ (*p* < 0.05).

## Data Availability

Data will be made available shared upon request from the corresponding author.

## Supplementary Materials

The following supporting information can be downloaded at: https://www.mdpi.com/article/10.3390/ani12182408/s1. Supplementary Table S1: Cytokine levels in piglets fed insoluble or soluble insoluble gruel creep feed diets, measured before (D22) and after weaning (D31), compared with non-creep-fed controls. One pig per litter was sampled at weaning (D22) or at one week post-weaning (D31). Supplementary Figure S1: Mini transition feeder. Supplementary Figure S2: Video-recorded feeding behavior of suckling pigs supplemented with insoluble or soluble gruel creep feed observed at 14 and 23 days of age. Means represent the time that piglets occupied feeder space, n = 6 litters/diet.
