# Supplementary material for: Gruel Creep Feeding Accelerates Growth and Alters Intestinal Health of Young Pigs
Source: Animals (Basel). 2022 Sep 14;12(18):2408. doi: 10.3390/ani12182408 (PMC9495199; doi:10.3390/ani12182408)
Supplement: Supplementary file 1 [file animals-12-02408-s001.zip › animals-1899616-supplementary.pdf]

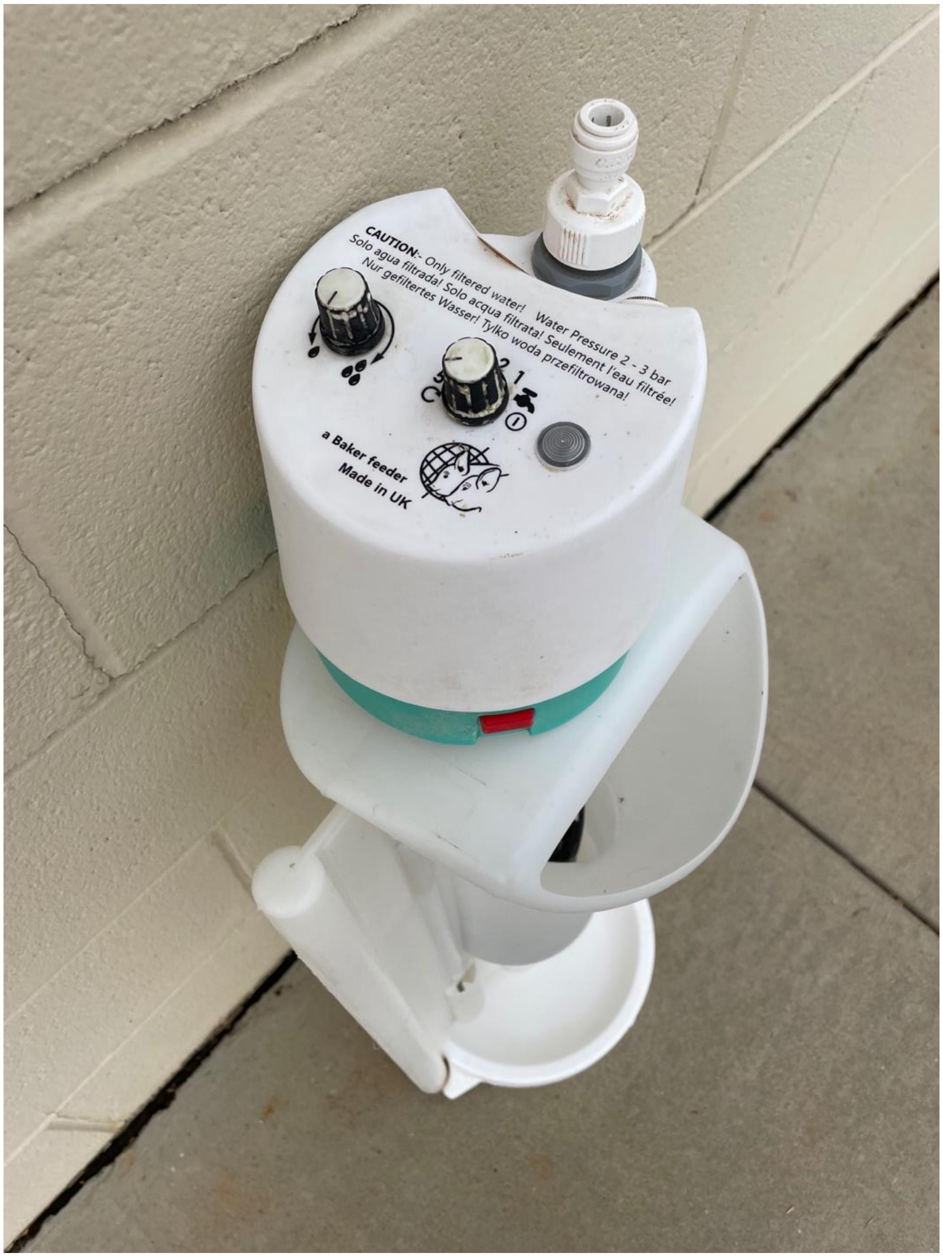

Supplementary Figure S1. Minitransition Feeder used to deliver gruel creep feeds

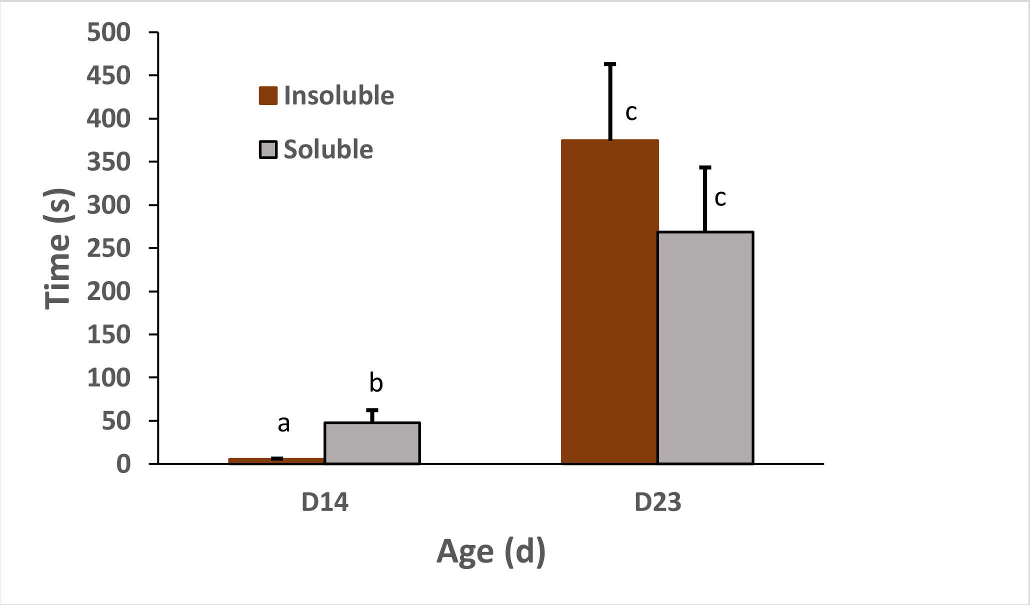

Supplemental Figure S2. Video-recorded feeding behavior of suckling pigs supplemented with insoluble or soluble gruel creep feed observed at 14 and 23 days of age. Means represent the time that piglets occupied feeder space, n = 6 litters/diet. [a, b, c] Bars within an age lacking a common superscript differ, $p < 0.05$.

Supplementary Table S1. Cytokine levels in piglets fed soluble or insoluble gruel creep feed diets, measured before (D22) and after weaning (D31) compared with non-creep-fed controls[1]

	Treatment							P > F		
Items (pg/ml)	Control		Insoluble		Soluble		SEM	Trt[2]	Age	T X A[3]
	D20	*D31*	*D20*	*D31*	*D20*	*D31*				
GM-CSF	72	21	75	47	37	39	30.7	0.76	0.32	0.69
IFNλ	8,543	5,627	6,117	5,346	6,703	6,533	967	0.38	0.11	0.34
IL-1α	43[a]	12[b]	13[b]	9[b]	11[b]	45[a]	10.5	0.19	0.99	0.02
IL-1β	541[a]	118[b]	133[b]	119[b]	104[b]	494[a]	103	0.12	0.86	0.01
IL-1ra	477	483	404	566	302	1,004	172	0.53	0.05	0.12
IL-2	370[a]	97[c]	133[abc]	90[c]	102[bc]	290[ab]	85	0.35	0.54	0.04
IL-4	1,169[abc]	204[c]	323[bc]	209[c]	228[c]	1,372[ab]	375	0.35	0.95	0.03
IL-6	174[a]	45[b]	54[b]	44[b]	49[b]	102[ab]	35.8	0.26	0.33	0.05
IL-8	121	99	103	113	92	106	13.6	0.70	0.97	0.35
IL-10	915[a]	274[b]	280[b]	213[b]	236[b]	604[ab]	201	0.24	0.50	0.06
IL-12	439	603	458	842	504	650	102	0.46	0.01	0.44
IL-18	2,291[a]	914[bc]	926[bc]	767[c]	912[bc]	2,025[ab]	417	0.17	0.68	0.02
TNFα	382	112	218	158	150	184	127	0.81	0.35	0.49

[1]One pig per litter was sampled at weaning (D22) or at one-week post-weaning (D31).

[2]Treatment.

[3]Treatment × Age

[abc]Means within a row lacking a common superscript differ ($P < 0.05$)
